# Antioxidant effect of grape seed extract corrects experimental autoimmune encephalomyelitis behavioral dysfunctions, demyelination, and glial activation

**DOI:** 10.3389/fimmu.2022.960355

**Published:** 2022-08-17

**Authors:** Maha Mabrouk, Mohamed El Ayed, Amélie Démosthènes, Youssef Aissouni, Ezzedine Aouani, Laurence Daulhac-Terrail, Meherzia Mokni, Mélina Bégou

**Affiliations:** ^1^ Université Clermont Auvergne, INSERM 1107, Neuro-Dol, Clermont-Ferrand, France; ^2^ Université Clermont Auvergne, Faculté de Pharmacie, Clermont-Ferrand, France; ^3^ Laboratoire de substances bioactives, Centre de Biotechnologie, Technopole de Borj Cedria, Hammam-Lif, Tunisia; ^4^ Faculté des Sciences de Tunis (FST), Université de Tunis el Manar (UTM), Tunis, Tunisia

**Keywords:** multiple sclerosis, experimental autoimmune encephalomyelitis, grape seed extract, oxidative stress, therapeutic approach, mice

## Abstract

**Background and purpose:**

Multiple sclerosis (MS), a multifactorial autoimmune disease of the central nervous system (CNS), is characterized by demyelination and chronic inflammation, as well as axonal and neuronal loss. There is no cure for MS, and despite a significant improvement in the therapeutic management of patients during the last 20 years, some symptoms are still resistant to treatment, and the evolution of the disease to progressive form seems still ineluctable. The etiology of MS is complex and still not fully understood. However, inflammation is a major driver of physiopathology and oxidative stress contributes to CNS lesions and promotes existing inflammatory response. Plant polyphenols are endowed with many therapeutic benefits through alleviating oxidative stress and inflammation, thus providing neuroprotection in MS. We presently evaluated the curative effect of grape seed extract (GSE) in an experimental autoimmune encephalomyelitis (EAE) mouse model of MS.

**Experimental approach:**

Six-week-old C57Bl/6J females were subjected to the EAE paradigm (using myelin oligodendrocyte glycoprotein peptide fragment (35-55), complete Freund’s adjuvant, and pertussis toxin) and then chronically treated with GSE from day 10 to day 30 post-induction. Clinical score and body weight were monitored daily, while evaluation of sensitive, motor, cognitive, and anxiety-related behaviors was performed weekly. Then, the GSE effect was evaluated on whole brain and spinal cord samples through the evaluation of oxidative stress damage, antioxidant capacities, myelin alteration, astroglial and microglial proliferation, and sirtuin expression.

**Key results:**

Grape seed extract curative chronic treatment corrected the clinical course of EAE, as well as the mechanical hypersensitivity, and avoided the development of EAE mouse thermal cold allodynia. The neuropathological evaluation showed that GSE reduced oxidative stress in the brain and spinal cord by decreasing the lipid and protein oxidation through correction of the three main antioxidant enzyme activities, namely, superoxide dismutase, catalase, and glutathione peroxidase, as well as restoring normal myelin protein expression and correcting microglial and astroglial protein overexpression and sirtuin downregulation.

**Conclusion and implications:**

These data strongly support GSE as an effective therapeutic approach in MS treatment.

## 1 Introduction

Multiple sclerosis (MS) is the most common chronic inflammatory and demyelinating disease of the central nervous system (CNS), affecting 2–3 million people worldwide (1, 2). It is a multifactorial disease with a complex and still imperfectly known etiology, whose main pathological hallmark is demyelinated lesions in CNS white matter, associated with inflammation, edema, axonal damage, and neurodegeneration (3). Many studies suggest oxidative stress as an important contributor to MS etiology, progression, and clinical symptoms. One common feature of oxidative stress is an altered oxidant/antioxidant balance leading to an excessive level of reactive oxygen species (ROS) (3, 4).

Depending on the location of the demyelinating lesions, a wide range of neurological symptoms, including motor, sensory, and cognitive impairments, can be observed in MS patients (1). One of the most frequent non-motor symptoms is chronic neuropathic pain observed in approximately 60% of MS patients (5). Neuropathic pain dramatically reduces a patient’s quality of life due to its distressing nature and persistent presence even in the early stages of the disease (6). There is no cure for MS, and despite a significant improvement in the therapeutic management of patients during the last 20 years, some symptoms (including pain) are still resistant to treatment (7). Furthermore, the evolution of the disease to a progressive form seems still ineluctable (2); thus, there is an urgent need for therapeutic innovation.

In recent years, numerous evidence suggests that plant polyphenols could have therapeutic benefits by alleviating oxidative stress and providing substantial neuroprotection in MS (8). Grape seed extract is a complex mixture of polyphenolics comprised of flavonoids and proanthocyanidins, which are the most abundant class of polyphenols classified as monomers of catechin and epicatechin along with complex oligomers (9, 10). In preclinical experiments, grape seed extract (GSE) exhibited numerous beneficial effects on various organs including the liver, heart, lung, and brain, thanks to its anti-inflammatory, antioxidant, and multi-organ protective properties (11–13). In the present work, we choose to evaluate the effect of chronic curative treatment with GSE in an experimental autoimmune encephalomyelitis (EAE) rodent model, which is routinely used to elucidate the mechanistic basis of MS and the putative therapeutic approaches. As the EAE model shares many clinical and neuropathological features with those observed in patients (14, 15), we choose an EAE model of mice in which chronic pain could be evaluated (16).

Grape seed extract effect was first, evaluated during the clinical course of the disease, through sensitive, motor, cognitive, and anxiety-related behaviors. Second, the GSE effect was evaluated on the brain and spinal cord samples through their ability to cope with oxidative stress damage (lipid and protein oxidation), loss in antioxidant capacities (enzymatic activity and protein expression of superoxide dismutase (SOD), catalase (CAT), and glutathione peroxidase (GPx)), myelin alteration, astroglial and microglial proliferation, and sirtuin expression.

## 2 Material and methods

### 2.1 Chemicals and drugs

The chemicals and drugs used for EAE induction were myelin oligodendrocyte glycoprotein peptide human fragment (MOG_35–55_ MEVGWYRPPFSRVVHLYRNGK), complete Freund’s adjuvant (CFA; heat-killed *Mycobacterium tuberculosis* 1 mg/ml) and pertussis toxin (PTX) purchased from Sigma-Aldrich (Darmstadt, Germany), ethanol and acetone purchased from Prolabo (Laboratoires humeau, La Chapelle-sur-Erdre, France), and sterile 0.9% sodium chloride (NaCl; CDM Lavoisier, Paris, France).

For biochemistry of oxidative stress and Western blotting analysis, the chemicals and drugs used were phosphate-buffered saline pH = 7.4 (PBS; Gibco, Grand Island, NY, USA), acetate and ethanol purchased from Prolabo (Laboratoireshumeau, La Chapelle-sur-Erdre, France), 2,6-di-*tert*-butyl-4-methylphenol (BHT), trichloroacetic acid (TCA), 2-thiobarbituric acid (TBA), 1,1,3,3-tetrathoxypropane (TMP), dinitrophenylhydrazine (DNPH), hydrochloric acid (HCl), hydrogen peroxide solution, sodium azide, ethylenediaminetetraacetic acid (EDTA), β-nicotinamide adenine dinucleotide phosphate sodium salt reduced (NADPH), SOD, CAT, glutathione reductase from baker’s yeast, reduced glutathione (GSH), GPx, bromophenol blue, and mercaptoethanol, all purchased from Sigma-Aldrich (Darmstadt, Germany).

### 2.2 Experimental autoimmune encephalomyelitis induction and curative chronic grape seed extract treatment

#### 2.2.1 Animals

Experiments were carried out in accordance with the European Communities Council Directive of 22 September 2010 (2010/63/UE) and the relative French law (Decree No. 2013-118 of 1 February 2013) with the approval of the local ethic committee (APAFIS-4306). They conformed to the ethics guidelines of the International Association for the Study of Pain (17) and were presented according to the ARRIVE 2.0 (18).

One hundred twenty female C57BL/6J mice at 4 weeks of age purchased from Janvier Laboratories (Le Genest Saint Isle, France) were used. Mice were housed six per cage (three CTL and three EAE) on standard sawdust in a temperature-controlled environment (22°C ± 2°C) under a 12:12-h light/dark cycle (light from 7:00 a.m. to 7:00 p.m.), with *ad libitum* access to food and water.

All tests took place during the light phase: between 8:00 and 12:00 a.m. for the von Frey, rotarod, and open field, and between 2:00 and 6:00 p.m. for elevated plus maze (EPM), hot plate, Y-maze, and acetone evaporation tests. Throughout the study, EAE scores and body weight were daily evaluated.

#### 2.2.2 Experimental autoimmune encephalomyelitis induction and assessment

After 2 weeks of habituation in our animal facility, following the protocol described by Olechowski et al. (2009) (using specifically female mice to observe sensitive phenotype), mice were immunized using 100 μl containing 50 μg of MOG35-55 emulsified in CFA (to 0.5 mg/ml CFA final concentration) administrated in two subcutaneous injections (50 μl each) in both flanks. Intraperitoneal injection of 300 ng of PTX dissolved in 200 μl of 0.9% NaCl was administered just after the subcutaneous injections (D0) and 48 h later (D2). Control animals (CTL) were injected with CFA (0.5 mg/ml) and PTX (300 ng) only. To prevent experimental bias, an independent experimenter (M. B.) different from that performing the rest of the study (M. M.) realized the EAE induction.

Mice were daily monitored for clinical scores and body weight in a blinded manner. For clinical scores determination, we used the scale point established by Olechowski et al. (2009) and graded it according to the following scale. Grade 0, normal mouse; grade 1, flaccid tail; grade 2, mild hindlimb weakness with quick righting reflex; grade 3, severe hindlimb weakness with slow righting reflex; and grade 4, hindlimb paralysis in one hindlimb or both. The EAE disease was considered present for clinical scores ≥1, and clinical scores ≤0.5 indicated no disease.

#### 2.2.3. Chronic curative grape seed extract treatment

Grape seeds were selected from a grape cultivar of *Vitis vinifera* (Carignan) grown in northern Tunisia (Grombalia). Briefly, seeds were air-dried, grounded with an electric mincer into a fine powder, and kept at room temperature (25°C) and in the dark in a glass bottle for further use. Before *in vivo* use, GSE composition has been evaluated as follows. Extraction of bioactive compounds i.e., polyphenolics, lipids, and sugars, was realized after suspending 400 mg of dry powder into 8 ml of 10% ethanol (w/2v) in the dark. Vigorous stirring and vortexing were followed by sonication for 3 min, allowing optimal extraction. The mixture was then centrifuged at 4°C (10,000 rpm for 15 min), and the supernatant containing bioactive compounds was analyzed by gas chromatography–mass spectrometry (GC-MS) according to (19). Briefly, polyphenolics and sugars were determined using the trimethylsilyl (TMS) derivatization method. For lipid phase analysis, extracts were methylated with sodium methylate in methanol, and samples were analyzed by GC/MS (5975C inert MSD with a Triple-Axis Detector; Agilent Technologies, Waldbronn, Germany). Quantification was made using an internal standard of d-myo-inositol for phenolic and aqueous phases and decane for the lipid phase. Concentrations were determined by plotting the concentration ratio against the standard area ratio (**Table 1**).

**Table 1:** Summary table of tests performed by cohorts.

Tests	Cohort 1 (n = 6/group)	Cohort 2 (n = 6/group)	Cohort 3 (n = 6/group)	Cohort 4 (n = 6/group)
**General observations**	Body weight EAE scoring	Body weight EAE scoring		
**Motor**	Rotarod	Rotarod	Open field	Open field
**Sensitive**	Hot plate Acetone paw test	Hot plate Acetone paw test	Von Frey Chaplan	Von Frey Chaplan
**Cognitive**	EPM	EPM	Y-maze	Y-maze

Body weight, EAE scoring, and von Frey Chaplan have been determined in all four cohorts, but we chose to show in this article only data from two cohorts highly representative of the other ones. EAE, experimental autoimmune encephalomyelitis; EPM, elevated plus maze.

In order to evaluate the chronic curative effect of GSE, the treatment started on day 10 (D10) post-immunization when the neurological score began to increase significantly and continued until D30. Daily GSE-treated mice received intraperitoneal injection of 200 µl of freshly prepared supernatant (as previously described for the evaluation of GSE composition) corresponding to 500 mg/kg body weight dosage per day. As control, vehicle-treated mice received a daily 200 µl intraperitoneal injection of 10% ethanol solution. Five cohorts (groups of animals immunized at the same time and evaluated using the same tests) were used for this study (four cohorts for behavioral evaluation and one for D20 biochemical sampling). The D30 biochemical samples obtained at the end of the behavioral experience and not used in this study will be included in future studies. Each cohort included 12 CTL and 12 EAE mice, randomly allocated to four groups of six animals: CTL + vehicle, EAE + vehicle, CTL + GSE, and EAE + GSE.

### 2.3 Behavioral evaluation

**Figure 1:**
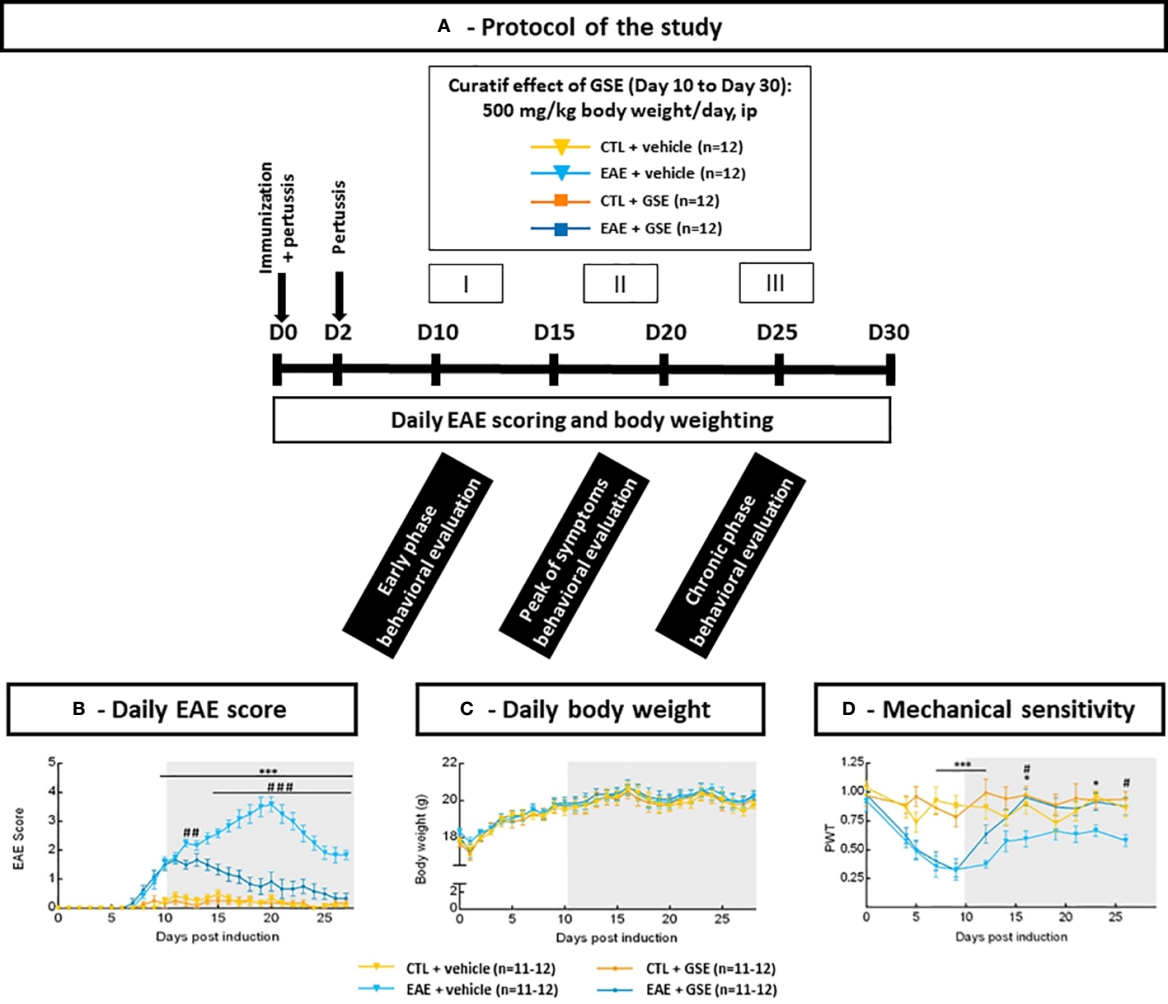
Longitudinal monitoring of clinical scores, body weight, and mechanical sensitivity in experimental autoimmune encephalomyelitis mice (EAE) and their controls (CTL) after curative treatment with grape seed extract (GSE) or vehicle. **(A)** Schematic representation of experimental autoimmune encephalomyelitis mice (EAE) induction, assessments, and curative chronic treatment with grape seed extract (GSE). **(B)** EAE clinical score was daily graded based on physical observation defined by Olechowski et al. (2009), ranging from 0 (normal behavior) to 4 (hindlimb paralysis in one hindlimb or both). Results are expressed as means ± SEM. **(C)** Daily evaluation of body weight. Results are expressed as means ± SEM. **(D)** Longitudinal monitoring of mechanical sensitivity using the von Frey test. Mechanical sensitivity was expressed as the mean of paw withdrawal threshold (PWT) ± SEM for each time point. Shadowed area represents times of evaluation after GSE treatment induction. Statistical analysis was performed using two-way ANOVA followed by post-hoc Sidak’s test: *p < 0.05, ***p < 0.001 EAE + vehicle vs CTL + vehicle and #p < 0.05, ##p < 0.01, ###p < 0.001 EAE + GSE vs EAE + vehicle.

Behavioral evaluation was realized during the early phase of the disease (D10–D12), the peak phase of symptoms (D17–D19), and the chronic phase of the disease (D24–D26) except for the von Frey test realized every 2 to 4 days (**Figure 1A**). The number of tests for each animal was limited to four to seven according to the severity of the behavioral procedures, and as a result, four cohorts of mice were necessary for this study (**Table 1**).

Body weight, EAE scores, and von Frey evaluation were determined in all four cohorts, but only compiled results from two highly representative cohorts are consigned to the data. All behavioral assessments were blindly conducted by an experimenter not aware of the immunization status.

#### 2.3.1 Evaluation of pain sensitivity

*Mechanical sensitivity* was evaluated using the von Frey test according to the up-and-down method of Dixon (1980) and adapted by Chaplan et al. (1994) (20, 21). Mice were placed on an elevated mesh platform (allowing access to the paws) in individual plastic boxes (3.5 cm long × 8 cm wide × 14 cm high). Before measurement of the paw withdrawal threshold (PWT), mice were habituated for 45–60 min to the apparatus. Stimulation was applied using the up-and-down method. Calibrated von Frey filaments ranging from 0.02 to 1.4 g (BiosebAesthesio, Chaville, France) were applied perpendicularly to the right hindpaw until reaching a slight buckling during 3–5 s. A positive response corresponds to a paw withdrawal, flinching, or licking. The PWT was determined as previously described by Dixon (20).

*Heat hyperalgesia* was assessed using the hot plate test (22), evaluating the reaction threshold to a high-intensity heat stimulus, which is considered an index of peripheral pain response. Mice were placed on a square metal surface (model-DS 37, Ugo Basile, Gemonio, Italy) and heated to a temperature of 52°C or 56°C without any restriction of movement. Different nocifensive responses can be elicited by the mice: licking, shaking hindpaw, or jumping. As soon as the first nocifensive response was observed, the timer was stopped, and the mouse was immediately removed from the hot plate. Data validation requires two stable latencies of less than 1 s of difference with a maximum of four trials performed per mouse. Data not fulfilling this validation criterion were excluded. To prevent paw injury, cut-off latencies of 30 s (for 52°C) and 15 s (for 56°C) were used.

*Cold allodynia* was evaluated using the acetone evaporation test adapted from Chen et al. (2018) (23). To allow full access to the paws, mice were placed on an elevated mesh platform in individual plastic boxes (3.5 cm long × 8 cm wide × 14 cm high) and then were allowed to acclimatize to the set-up for 30–45 min. A 20-μl drop of acetone was laid on the plantar surface of the hindpaw without touching the skin with the dropper tip, and the response elicited by the mouse was observed during 60 s. Responses to acetone were scored as follows: 0, no response; 1, quick withdrawal, flick, or stamp of the paw; 2, prolonged withdrawal or repeated stamping or flicking of the paw; 3, licking of the paw; and 4, jumping. The nocifensive score was the sum of all the responses evoked during the period of 60 s post-acetone application. For each mouse, acetone was applied alternately three times to each hindpaw, and the nocifensive scores obtained for the six stimulations were averaged for each mouse given that we did not target one particular side of the CNS.

#### 2.3.2 Motor evaluation

*Spontaneous locomotion activity* was evaluated using the open-field test. Freely moving mice were placed in the center of a white polyvinyl chloride open-field apparatus (50 cm long × 50 cm wide × 45 cm high) for 15 min. The video tracking software (Viewpoint, Lyon, France) allowed us to determine virtual areas: a central square (30 cm long) and a peripheral zone (corridor of 10 cm wide). Total distance travelled as an index of locomotion and time spent in the central area as an index of anxiety-related behavior were recorded using the video tracking system.

*Motor coordination* was determined using a standard mouse rotarod (TSE, Homburg, Germany) using the standard operating procedures described by the EUMORPHIA group program (24). Briefly, a training phase identifying mice able to stay on the rod at 4 rpm for 60 s was first realized. Successful mice then underwent a test phase comprising four trials with a 15-min intertrial interval. In each trial (T1–T4), mice were placed on the rod rotating at 4 rpm, the timer was started, and the rod accelerated from 4 to 40 rpm in 300 s. The latency to fall off the rod was determined automatically except for ‘passive rotation’ (mouse held onto the rod completing a full rotation) for which the timer was manually stopped.

#### 2.3.3 Cognitive evaluation

*Working memory* was evaluated using the Y-maze according to the standard operating procedure described by the EUMORPHIA group program (24). The maze was made of three equal arms (40 cm long × 10 cm wide × 16 cm high) in black polyvinyl chloride radiating at 120° from each other, providing the shape of Y. Mice were placed at the end of one arm of the maze, facing the wall, and allowed to explore freely for a 10-min session. Latency to leave the first arm, total number, and sequence of entries into each arm were scored for each mouse. An arm entry was counted when the mouse had all four paws inside the arm. If a mouse completed fewer than eight arm entries during the entire session, its data were excluded from further analysis. Spontaneous alternation was defined as entries into all three arms on three consecutive choices. The alternation score (%) represents an index of working memory and was calculated as % Alternation = 100 × [Number of alternations/(Total arm entries − 2)]. The total number of arm entries was also evaluated as an index of locomotion and the time spent to enter in the first new arm as an index of anxiety-related behaviors.

*Anxiety* was assessed using the EPM testing the natural conflict between the tendency of mice to explore a novel environment and their tendency to avoid a brightly lit, elevated, open area (25). The EPM was made of Plexiglas (black floor and walls) and comprised two open arms and two closed arms (37 cm long × 6 cm wide) that extended from a central platform (6 cm × 6 cm). To increase the anxiogenic value of the open arms, the maze was elevated at 50 cm above floor level. The mouse was placed on the central platform, facing one of the enclosed arms, and allowed to explore freely for 10 min. With the use of the video tracking system (Viewpoint, Lyon, France), the time spent in the open arms was calculated. Mice completing fewer than eight arm entries within 10 min were excluded from further analysis.

### 2.4 Oxidative stress biochemical analysis

#### 2.4.1 Tissue preparation and homogenization

Mice from two different cohorts were sacrificed at D20 (during the peak of symptoms) and D30 (during the chronic phase of the disease), and the whole encephalon and spinal cord were rapidly removed, snap-frozen in liquid nitrogen, and next stored at −80°C.

For the biochemical study, six brains (telencephalon and diencephalon) and six spinal cords for each group were lysed in ice-cold PBS buffer, supplemented with a complete protease inhibitor cocktail (Roche, Basel, Switzerland) using piston sonicator 20 kHz with optimal amplitude (Fisher brand™ model 505, Thermo Fisher Scientific, USA). Each sample was subjected to 3 cycles of sonication (30 s) separated by a 10-s stop. Homogenates were then centrifuged for 15 min at 13,000 rpm at 4°C, and supernatants were aliquoted and stored at −80°C until analysis, after total protein determination using a bicinchoninic acid (BCA) protein assay kit (Thermo Fisher Scientific, Inc., Waltham, MA, USA) according to manufacturer’s instructions.

#### 2.4.2 Brain and spinal cord lipid peroxidation

Lipid peroxidation was determined spectrophotometrically following measurement of malondialdehyde (MDA) level (nmol/mg protein) based on the reaction with TBA according to the method described by Draper et al. (1990), slightly modified (26). Briefly, 137.5 µl of tissue homogenate (protein content 50 µg/ml) was mixed in a 96-well plate (Nunc-Immuno MicroWell) with 8.3 µl of BHT 3.5 mM, 62.5 µl of TCA 50%, and 41.5 µl of TBA 1.25% (m/v) and incubated at 60°C for 60 min. The reaction was stopped by cooling the plate into ice for 5 min, and absorbance of the resulting pink chromophore was measured (in triplicate) at 532 nm using a UV–Visible microplate spectrophotometer (Epoch BioTek Instruments, Agilent Technologies, Santa Clara, CA, USA). As a standard for the calibration curve, TMP was used in the range of 0 to 6.5 μM.

#### 2.4.3 Brain and spinal cord protein carbonylation

Oxidative damage of proteins was evaluated by quantifying protein carbonylation according to Levine et al. (1990), with slight modifications (27). The total protein-bound carbonyl content was determined by derivatizing the protein carbonyl adducts with DNPH, which forms a stable dinitrophenyl hydrazine product. Tissue homogenate measuring 100 µl (protein content 0.6 mg/ml) was mixed with 400 µl of DNPH solution (10 mM in 2.5 M of HCl) for 1 h at 37°C in the dark with mixing every 10 min, followed by protein precipitation with 250 µl of TCA 30% and mixture centrifugation at 11,000 rpm to obtain a pellet. DNPH was removed after three washings of the pellet using 500 µl of ethyl acetate/ethanol (1:1, v/v) solution (each followed by 5-min centrifugation at 11,000 rpm and 4°C to reobtain the pellet). Afterward, the pellet was redissolved into 600 µl of guanidine hydrochloride 6 M prior to absorbance reading (in triplicate) at 375 nm using a UV–Visible microplate spectrophotometer (Epoch BioTek Instruments, Agilent Technologies, USA). The level of DNPH derivatized proteins was determined using the molar extinction coefficient of 22.000 M^−1^ cm^−1^, and protein carbonylation level was expressed as nanomoles of carbonyl residues per milligram of total proteins (nmol/mg protein).

#### 2.4.4 Antioxidant enzyme activities in the brain and spinal cord

SOD (E.C.1.15.1.1) activity was determined using the Sigma-Aldrich kit (19160-1KT-F, Sigma-Aldrich, Darmstadt, Germany) according to the manufacturer’s instructions. Briefly, SOD activity was measured using the highly water-soluble tetrazolium salt, WST-1 [2-(4-iodophenyl)-3-(4-nitrophenyl)-5-(2,4-disulfophenyl)-2H-tetrazolium, monosodium salt], which produces a water-soluble formazan dye upon reduction with superoxide anion. The 50% inhibition activity of SOD was determined by measuring the decrease in the absorption spectrum of WST-1 formazan. SOD activity was determined in 96-well plates (Nunc-Immuno MicroWell) with absorbance measurement at 450 nm using a UV–Visible microplate spectrophotometer (Epoch Bio-Tek Instruments, Agilent Technologies, Santa Clara, CA, USA). SOD activity was expressed in terms of percent of inhibition, and commercial SOD was used as a standard for calibration curve in the range of 0 to 200 U/ml. Assays were conducted in triplicate.

CAT (E.C.1.11.1.6) activity was determined by measuring the kinetics of H_2_O_2_ disappearance at 240 nm for 3 min (28) using a JENWAY 7305 spectrophotometer (Bibby Scientific, Cole Parmer, UK). The reaction mixture contained 10 µl of 1.25 mg/ml of protein mixed with 990 µl of 33 mM H_2_O_2_ in 50 mM of phosphate buffer pH 7 in a 1-ml final volume. Commercial CAT was used as a standard for the calibration curve in the range of 0 to 600 U/ml (R^2^ = 0. 9925). Results were expressed as mmolH_2_O_2_ consumed per min per mg protein (mmolH_2_O_2_/min/mg protein). Assays were conducted in triplicate.

GPx (EC.1.11.1.9) activity was determined by measuring the consumption of NADPH at 340 nm for 3 min (29, with slight modification) using a JENWAY 7305 spectrophotometer (Bibby Scientific, Cole Parmer, UK). Briefly, 20 µl of diluted homogenate tissue (protein concentration adjusted to 1.25 mg/ml) was mixed with potassium buffer 50 mM pH = 7, containing 2.5 mM of NaN_3_, 2.5 mM of EDTA, 2.5 mM of NADPH, 10 mM of GSH, and 6 µU/ml of glutathione reductase. After 1 h of incubation at 37°C, 100 µl of H_2_O_2_ 0.33 mM was added, and the reaction started. Commercial GPx was used as the standard for the calibration curve in the range of 0 to 0.25 U/ml. Results were expressed as mmolH_2_O_2_ per min per mg protein (mmolH_2_O_2_/min/mg protein). Assays were conducted in triplicate.

### 2.5 Western blotting analysis

Supernatants of the brain and spinal cord samples were also used for the determination of expression of some proteins of interest. After 5-min denaturation at 95°C in Laemmli loading buffer (bromophenol blue/mercaptoethanol), protein samples (10–30 µg) were subjected to sodium dodecyl sulfate–polyacrylamide gel electrophoresis (SDS–PAGE) on 10% polyacrylamide gels (Bio-Rad Laboratories, Hercules, CA, USA) and transferred to nitrocellulose membranes using a Trans-Blot Turbo transfer system (Bio-Rad Laboratories, Hercules, CA, USA). Membranes were then blocked in Tris-buffered saline (TBS) with 0.1% Tween (0.1% TBS-T) with 5% w/v non-fat dry milk or 5% w/v bovine serum albumin (BSA) for 1.5 h at room temperature and next incubated with primary antibodies overnight at 4°C. The primary antibodies used were anti-SOD2 (R&D Systems, Minneapolis, MN, USA; MAB 3419, 1/3,000), anti-SOD1 (Thermo Fisher Scientific, MA1-105, 1/1,000), anti-CAT (Cell Signaling Technology, Danvers, MA, USA; D5N7V, 1/1,000), anti-GPX1 (Invitrogen, Carlsbad, CA, USA; PA5-30593, 1/1,000), anti-glial fibrillary acidic protein (anti-GFAP; Millipore, Billerica, MA, USA; ab5804, 1/1,000), anti-ionized calcium binding adaptor molecule 1 (Iba-1, Invitrogen, PA5-27436, 1/1,000), anti-myelin basic protein (anti-MBP; Millipore, MAB 384, 1/500), anti-2′,3′-cyclic-nucleotide 3′-phosphodiesterase (CNPase, Millipore, MAB 326, 1/1,000), anti-sirtuin1 (SIRT1, Cell Signaling, 9475S, 1/500), anti-sirtuin2 (SIRT2, Cell Signaling, 12650, 1/1,000), anti-glyceraldehyde-3-phosphate dehydrogenase (GAPDH, Sigma-Aldrich, CB1001, 1/15,000), anti-β-tubulin (Cell Signaling Technology, 86298S, 1/1,000), anti-α-tubulin (Cell Signaling Technology, 2144, 1/1,000), and anti-β-actin (Sigma-Aldrich, A5441, 1/5,000). After 90-min incubation with corresponding horseradish peroxidase (HRP)-conjugated secondary antibodies [anti-mouse Ig G (P0447; DAKO, Santa Clara, CA, USA), anti-rabbit Ig G (Cell Signaling Technology, Danvers, USA; 7074S)], proteins were visualized using an enhanced chemiluminescence substrate mixture (Pico or Clarity Western ECL Substrate, Bio-Rad Laboratories, Inc., Hercules, CA, USA). Immunoblots were revealed using the ChemiDoc Touch Gel imaging system (Bio-Rad Laboratories, Hercules, CA, USA) and quantified using Image Lab software (Bio-Rad Laboratories, Hercules, Cam USA). Protein levels were normalized to the internal control α-tubulin, β-actin, or GAPDH.

### 2.6 Statistical analysis

All statistical analyses were carried out using GraphPad Prism 6 software (GraphPad Inc., San Diego, CA, USA). Equality of variance and normal distribution were evaluated before each analysis to determine the type of analysis to be performed: parametric or non-parametric.

For body weight and EAE score longitudinal monitoring, data were analyzed using a two-way analysis of variance (ANOVA) with groups (CTL + vehicle, EAE + Vehicle, CTL + GSE, and EAE + GSE) and day post-induction (DPI, D0–D30) as main factors and with DPI defined as a repetitive measure. *Post-hoc* comparisons were made with Tukey’s comparison test for multiple comparisons between groups for each DPI. For motor, sensitivity, and cognitive behavioral tests, data were analyzed using two-way ANOVA with groups (CTL + vehicle, EAE + Vehicle, CTL + GSE, and EAE + GSE) and day post-induction (DPI, D0–D30) as the main factors, with DPI being defined as a repetitive measure. As recommended by the statistical software, Tukey’s comparison test was used for multiple comparisons between groups for each time point.

For oxidative stress biochemical analysis and Western blotting analysis, statistical analysis was performed using one-way ANOVA followed by Tukey’s comparison test. To simplify the comparison between D20 and D30 results, data were expressed as the relative value (%) compared to CTL + vehicle group.

Results were presented as mean ± standard error of the mean (SEM) for behavioral study or as raw data + mean for the oxidative stress biochemical analysis and Western blotting analysis. A *p*-value <0.05 was taken as the statistical significance level.

## 3 Results

### 3.1 Grape seed extract composition

Gas chromatography–mass spectrometry analysis of GSE composition revealed that the most represented compound within the phenolic phase was epicatechin (35.21%) followed by catechin and gallic acid (**Table 2**). Within the lipid phase, the most abundant compound was glycerol monostearate (68.95%) followed by the 2-monostearin and glycine, *N*-methyl-*n*-propoxycarbonyl-, hexadecyl ester. Finally, within the aqueous phase, the most abundant compound was glucopyranoside (26.42%) followed by fructofuranose isomer 1 and sucrose.

**Table 2:** GSE main components.

Components	Relative abundance (%)
**Polyphenolics**	
Epicatechin	35.21
Catechin	34.41
Gallic acid	12.84
Quercetin derivative	5.73
Flavan-3-ol	2.25
Protocatechuic acid	2.20
Gallic acid ethyl ester	1.73
Tyrosol	1.03
Quercetin	1.01
**Lipids**	
Glycerol monostearate	68.95
2-Monostearin	15.48
Glycine, *N*-methyl-*n*-propoxycarbonyl-, hexadecyl ester	9.74
α-Monopalmitin	2.57
Squalene	1.34
**Sugars**	
Glucopyranoside	26.42
Fructofuranose isomer 1	17.31
Sucrose	11.78
β-d-Glucopyranose	7.30
Sorbitol	6.76
Unknown polysaccharide	6.39
Myo-inositol	3.40
Fructofuranose isomer 2	2.23
Trehalose	1.99
d-Psicose	1.64
Gulose	1.51
d-Allofuranose	1.00

GSE, grape seed extract.

### 3.2 Grape seed extract curative chronic treatment corrects clinical symptom development and respects body weight in experimental autoimmune encephalomyelitis mice

A significant increase in EAE score was observed from D9 to D28 post-induction (Tukey’s test, p < 0.0001 from D9 to D27). Curative GSE treatment reduced this clinical development as soon as D12 (Tukey’s test; D12, p < 0.01 and D14–D27, p < 0.0001) until recovering a score similar to CTL mice from D25 to sacrifice (Tukey’s test, D25 to D28 p > 0.05) (**Figure 1B**). Body weight was also monitored daily, and statistical analysis revealed no difference between groups (Tukey’s test, D0 to D28 p > 0.05) (**Figure 1C**). These data highlight that chronic curative GSE treatment corrects the development of the EAE clinical course without effect on body weight.

### 3.3 Grape seed extract treatment corrects experimental autoimmune encephalomyelitis mouse mechanical hypersensitivity and avoids the development of experimental autoimmune encephalomyelitis mouse thermal cold allodynia

**Figure 2:**
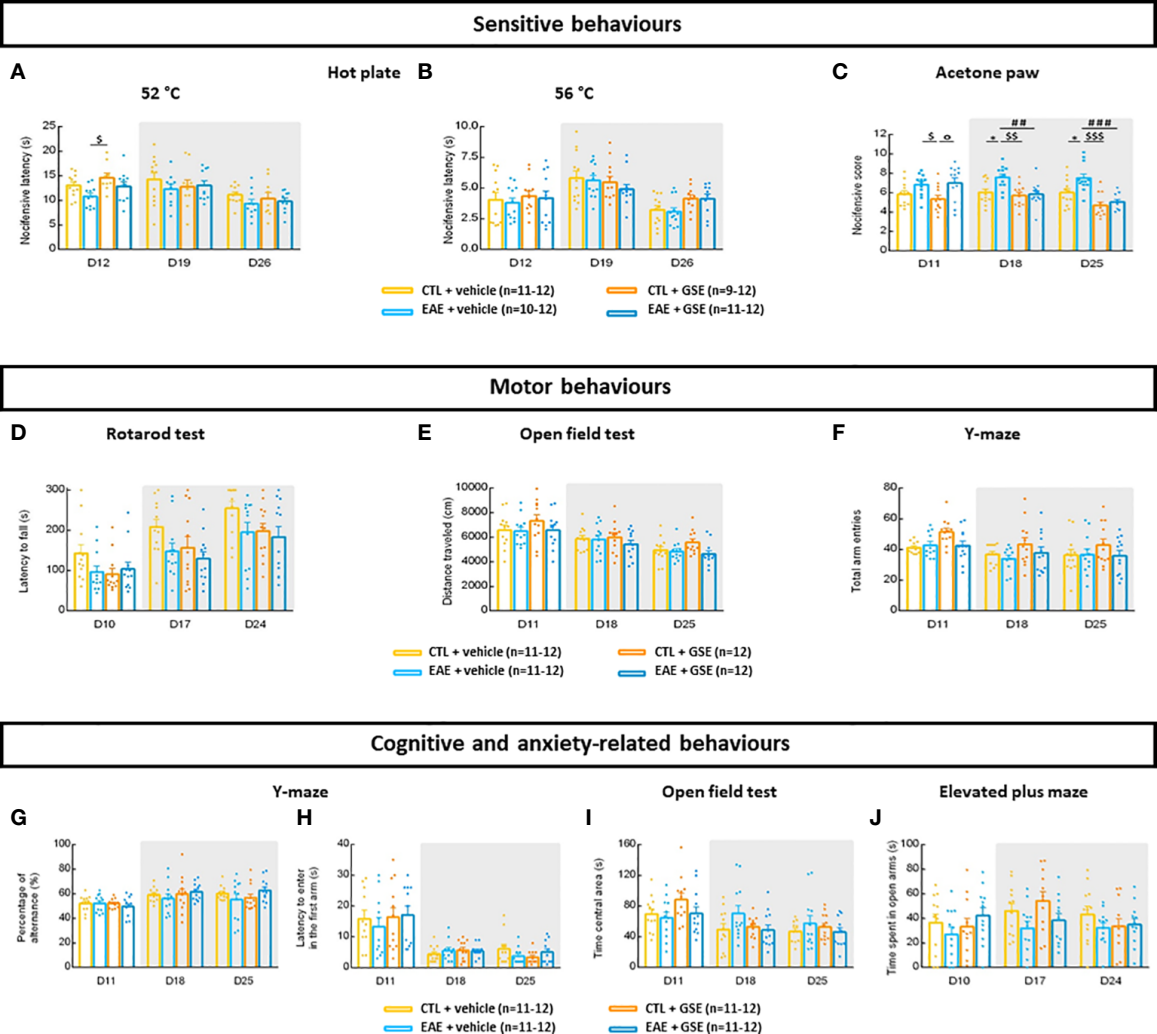
Evaluation of sensitive, motor, cognitive, and anxiety-related behaviors in experimental autoimmune encephalomyelitis mice (EAE) and their controls (CTL) after curative treatment with grape seed extract (GSE) or vehicle. **(A, B)** Evaluation of heat hyperalgesia using the hot plate test expressed as the mean time taken to observe a nocifensive behavior in mice exposed to a thermal plate maintained at 52°C **(A)** or 56°C **(B)**. Data are expressed as mean nocifensive latency ± SEM. **(C)** Evaluation of cold allodynia using the acetone paw test expressed as the mean number of nocifensive response ± SEM observed in mice after acetone drop deposition. **(D)** Evaluation of motor coordination using rotarod test, expressed as the mean latency to fall ± SEM for the four-rotarod sessions performed by each mouse. **(E)** Evaluation of spontaneous locomotor activity using the open field, expressed as the total distance travelled ± SEM for the overall 15-min session. **(F)** Evaluation of spontaneous locomotor activity using the Y-maze, expressed as the total number of arm entries ± SEM for the overall 10-min session. **(G, H)** Evaluation of working memory and anxiety-related behaviors using the Y-maze. The working memory was evaluated using the percentage of alternation ± SEM for the overall 10-min session **(G)** and anxiety-related behaviors were evaluated using the time spent to enter the first new arm ± SEM during the overall 10-min session **(H)**. **(I)** Evaluation of anxiety-related behaviors using the open-field test expressed as the time spent in central area ± SEM for the overall 15-min session. **(J)** Evaluation of anxiety-related behaviors using the elevated plus maze (EPM), expressed as the time spent in the open arms ± SEM for the overall 10-min session. Shadowed area represents times of evaluation after GSE treatment induction. Statistical analysis was performed using two-way ANOVA followed by post-hoc Sidak’s test: *p < 0.05 EAE + vehicle vs CTL + vehicle; ##p < 0.01, ###p < 0.001 EAE + GSE vs EAE + vehicle; $p < 0.05, $$p < 0.01, $$$p < 0.001 EAE + vehicle vs CTL + GSE and °p < 0.05 EAE + GSE vs CTL + GSE.

Evaluation of mechanical hypersensitivity revealed in EAE mice a significant decrease in PWT from D7 to D12 (Tukey’s test, D7–D12, p < 0.0001) as well as at D16 and D23 (Turkey’s test, p < 0.05). Curative GSE treatment corrected such hypersensitivity at D16 (the peak of symptoms) and D26 (chronic phase of the disease) (Turkey’s test, p > 0.05) (**Figure 1D**). Evaluation of thermal heat sensibility using the hot plate test revealed no statistical difference between groups at 52°C and 56°C (Turkey’s test, p < 0.05) (**Figures 2A, B**). Finally, when assessing cold allodynia using the acetone paw test, a significant increase in nocifensive score was observed in EAE + vehicle mice at D18 and D25 (Tukey’s test, p < 0.05) but not in EAE + GSE mice (Tukey’s test, p > 0.05) (**Figure 2C**). Taken together, these results show that chronic GSE treatment corrects the development of mechanical hypersensitivity and avoids the development of cold allodynia in EAE mice.

### 3.4 Grape seed extract and experimental autoimmune encephalomyelitis models have no effect on motor coordination, locomotion activity, working memory, and anxiety-related behaviors

Motor coordination, assessed by the latency to fall from the rotarod, showed no significant difference between groups whatever the day of testing (Tukey’s test, p > 0.05) (**Figure 2D**). Spontaneous locomotor activity was evaluated by the total distance travelled in the open-field test as well as the total arm entries in the Y-maze. Statistical analysis revealed no significant difference between groups (Tukey’s test, p > 0.05) (**Figures 2E, F**). Working memory, evaluated using the Y-maze, showed no significant difference between groups regarding the percentage of alternation (Tukey’s test, p > 0.05) (**Figure 2G**). Finally, anxiety-related behaviors were evaluated by 1) the time to enter the first new arm using the Y-maze test, 2) the time spent in the central zone using the open-field test, and 3) the time spent in the open arms using the EPM. In all three tests, no significant difference between groups was observed (Tukey’s test, p > 0.05) (**Figures 2H–J**). Taken together, these data suggest that GSE as well as our model of EAE mice had no effect on motor coordination, locomotion activity, working memory, and anxiety-related behaviors.

### 3.5 Grape seed extract treatment rescues brain and spinal cord oxidation in experimental autoimmune encephalomyelitis mice

**Figure 3:**
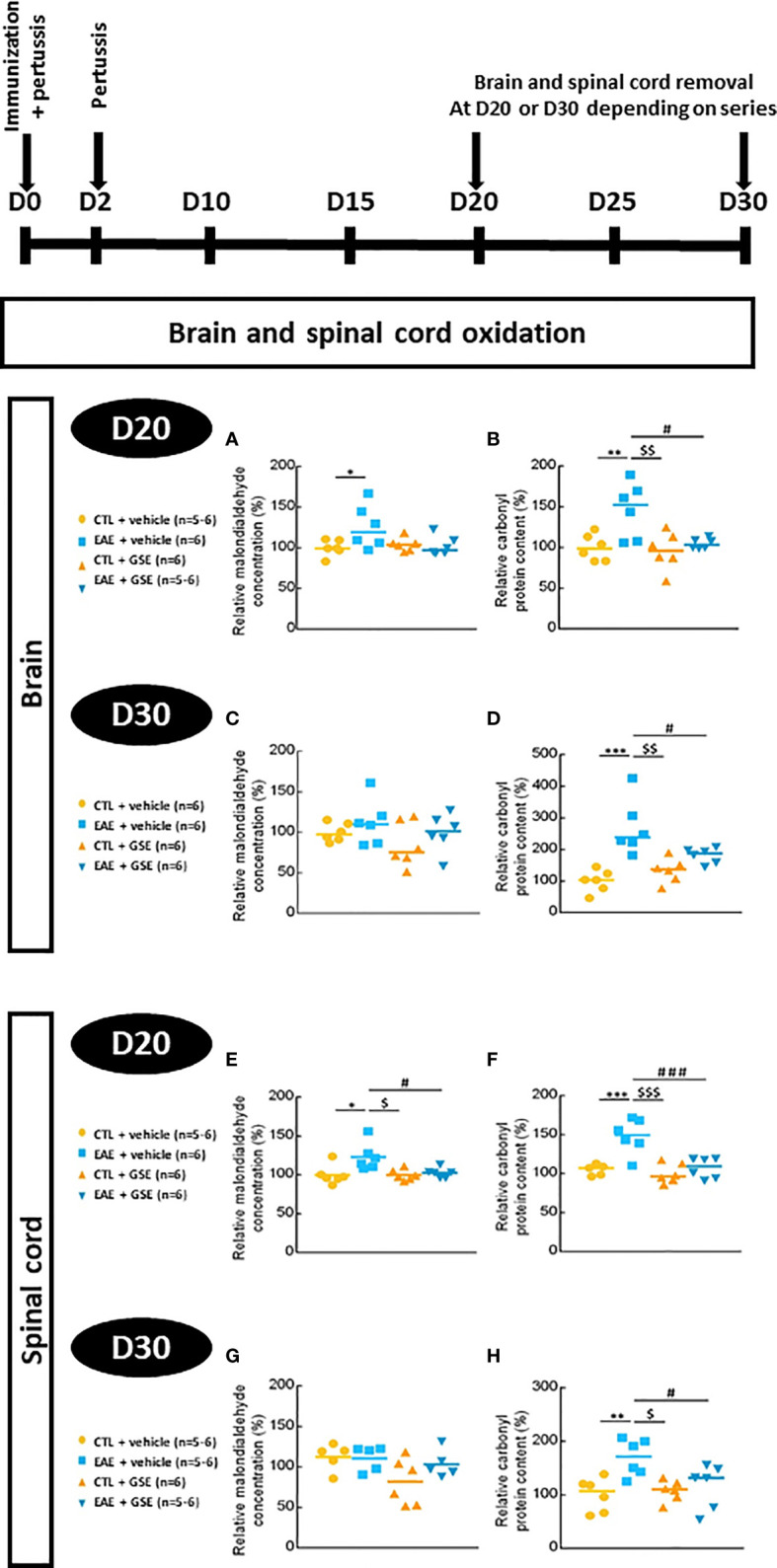
Level of lipid and protein oxidation in the brain and spinal cord of experimental autoimmune encephalomyelitis mice (EAE) and their controls (CTL) after curative treatment with grape seed extract (GSE) or vehicle. The lipid peroxidation level was measured by the estimation of relative malondialdehyde concentration compared to CTL + vehicle group, in the brain **(A, C)** and the spinal cord **(E, G)** at D20 **(A, E)** or D30 **(C, G)** post-induction. Oxidative damage to proteins was evaluated by quantifying the relative carbonyl protein content compared to CTL + vehicle group, in the brain **(B, D)** and the spinal cord **(F, H)** at D20 **(B, F)** or D30 **(D, H)** post-induction. Statistical analysis was performed using one-way ANOVA followed by Tukey’s multiple comparison test: *p < 0.05, **p < 0.01, ***p < 0.001 EAE + vehicle vs CTL + vehicle; #p < 0.05, ###p < 0.001 EAE + GSE vs EAE + vehicle and $p < 0.05, $$p < 0.01, $$$p < 0.001 EAE + vehicle vs CTL + GSE.

At D20, an increase of 20%–25% in MDA level was observed in EAE + vehicle brains and spinal cord (Tukey’s test, p < 0.05) but not in EAE + GSE (Tukey’s test, p > 0.05) (**Figures 3A, E**). At D30, during the chronic phase of EAE, there was no statistical difference in MDA between groups (Tukey’s test, p > 0.05) (**Figures 3C, G**).

In the brain, an increase in the content of carbonyl protein was observed in EAE mice (46% at D20 Tukey’s test, p < 0.01, and 149% at D30 Tukey’s test, p < 0.001), which was corrected upon GSE treatment (**Figures 3B, D**). Similarly, in the spinal cord, increased content of carbonyl protein was observed in EAE mice (69% at D20 Tukey’s test, p < 0.001, and 48% at D30 Tukey’s test, p < 0.01) also corrected upon GSE treatment (**Figures 3F, H**). These data suggest a corrective effect of GSE on the transitory increase in lipid peroxidation during the peak of symptoms and the long-lasting increase in protein oxidation in both the brain and spinal cord as soon as 10 days post-treatment.

### 3.6 Grape seed extract treatment restores brain and spinal cord antioxidant enzyme activities and protein expression

#### 3.6.1 Superoxide dismutase activity and protein expression

**Figure 4:**
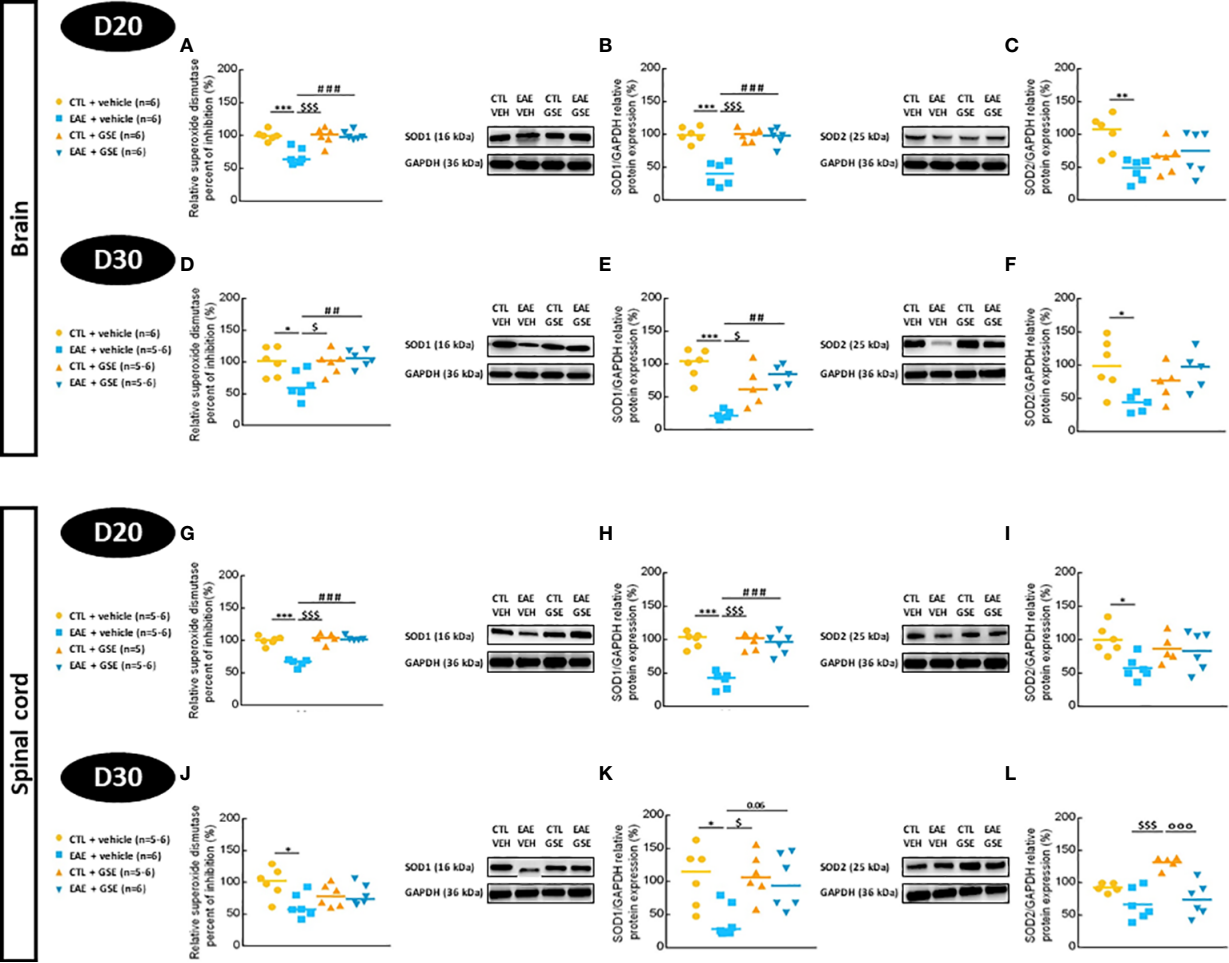
Evaluation of superoxide dismutase activity and protein expressions in the brain and spinal cord of experimental autoimmune encephalomyelitis mice (EAE) and their controls (CTL) after curative treatment with grape seed extract (GSE) or vehicle. Superoxide dismutase activity was expressed as the relative percent of inhibition (%) compared to CTL + vehicle group, in the brain **(A, D)** and the spinal cord **(G, J)** at D20 **(A, G)** or D30 **(D, J)** post-induction. SOD1/GAPDH protein expression was expressed as the relative protein expression (%) compared to CTL + vehicle group, in the brain **(B, E)** and the spinal cord **(H, K)** at D20 **(B, H)** or D30 **(E, K)** post-induction. SOD2/GAPDH protein expression was expressed as the relative protein expression (%) compared to CTL + vehicle group, in the brain **(C, F)** and the spinal cord **(I, L)** at D20 **(C, I)** or D30 **(F, L)** post-induction. For each protein level quantification, a representative Western blotting image of the target protein and loading control is proposed. Statistical analysis was performed using one-way ANOVA followed by Tukey’s multiple comparison test: *p < 0.05, **p < 0.01, ***p < 0.001 EAE + vehicle vs CTL + vehicle; ##p < 0.01, ###p < 0.001 EAE + GSE vs EAE + vehicle, $p < 0.05, $$$p < 0.001 EAE + vehicle vs CTL + GSE and °°°p < 0.001 EAE + GSE vs CTL + GSE.

At D20 and D30, SOD activity decreased in EAE mouse brains by 32% and 36%, respectively (Tukey’s test, p < 0.001 at D20 and p < 0.05 at D30) and was corrected by GSE (**Figures 4A, D**). Similar results were found in the spinal cord where SOD activity decreased in EAE mice (29% at D20 Tukey’s test, p < 0.001 and 36% at D30 Tukey’s test, p < 0.01) and was significantly corrected by GSE only at D20 (**Figures 4G, J**). To explain the decrease in total SOD activity, we suspected a decrease in the protein abundance of cytosolic SOD1 (Cu-Zn) and mitochondrial SOD2 (Mn-SOD). Western blotting analysis of brain extracts revealed a decrease in SOD1 (61% at D20 and 69% at D30, Tukey’s test, p < 0.001) (**Figures 4B, E**) and SOD2 (55% at D20 Tukey’s test, p < 0.01 and 24% at D30 Tukey’s test, p < 0.05) (**Figures 4C, F**) protein expressions in EAE mice. A significant effect of GSE was observed for SOD1 protein expression at D20 and D30 but only a trend for SOD2 protein expression. Western blotting analysis of spinal cord extracts revealed a decrease in SOD1 (68% at D20 Tukey’s test, p < 0.001 and 59% at D30 Tukey’s test, p < 0.05) (**Figures 4H, K**) and SOD2 (33% only significant at D20 Tukey’s test, p < 0.05) (**Figures 4I, L**) protein expressions in EAE mice. GSE restored a normal SOD1 protein expression in the spinal cord at D20 and D30 without a significant effect on SOD2 (**Figures 4H, K**). These data obtained in the brain and spinal cord suggest a decrease in SOD activity in EAE mice from the peak of symptoms to the chronic phase of the disease correlated to a decreased expression in SOD1 but to a lesser extent for SOD2 isoforms. Furthermore, the beneficial effect of GSE on SOD activity seems mostly related to an increase in SOD1 protein level.

#### 3.6.2 Catalase activity and protein expression

**Figure 5:**
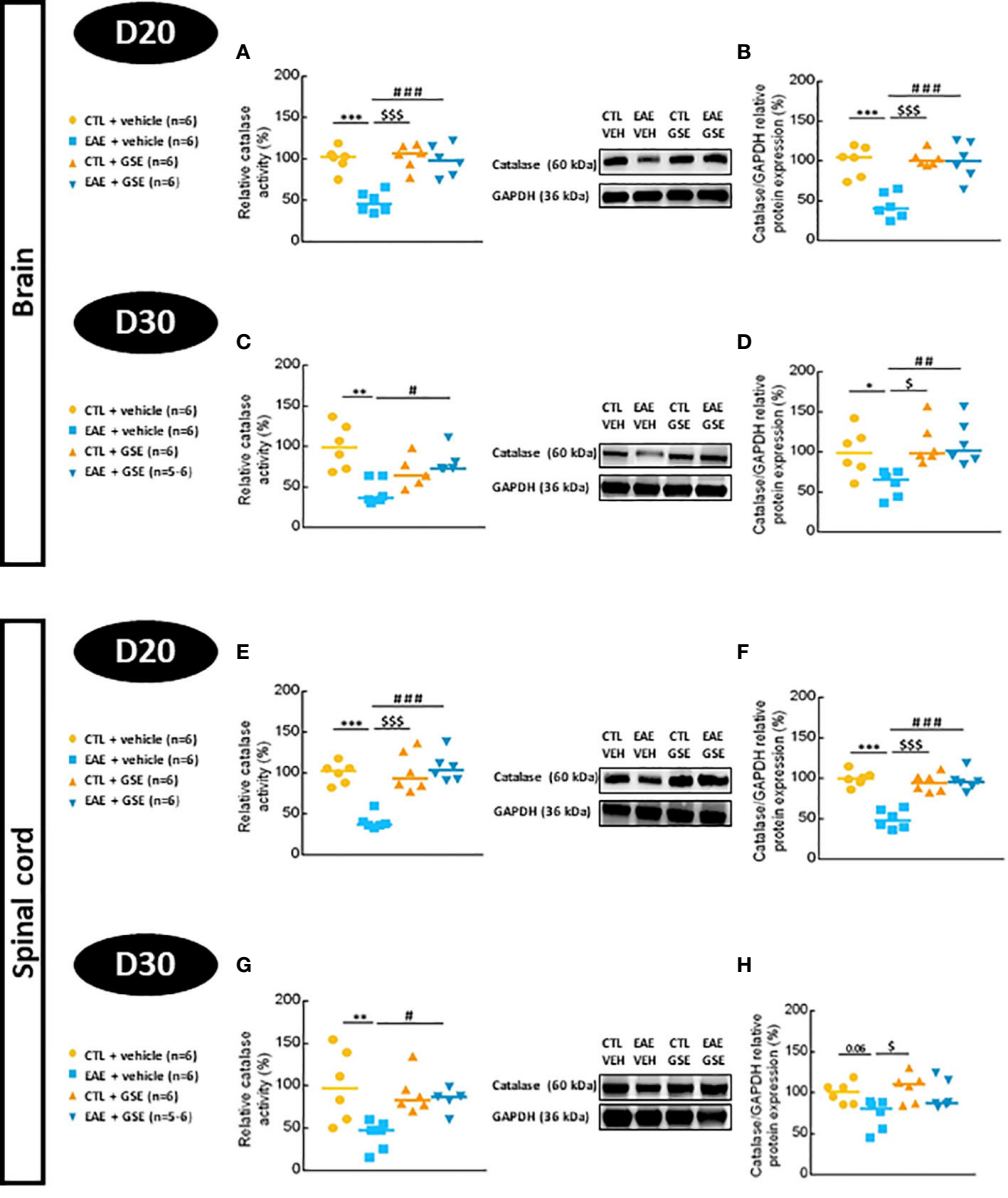
Evaluation of catalase activity and protein expression in the brain and spinal cord of experimental autoimmune encephalomyelitis mice (EAE) and their controls (CTL) after curative treatment with grape seed extract (GSE) or vehicle. Catalase activity was expressed as the relative activity (%) compared to CTL + vehicle group, in the brain **(A, C)** and the spinal cord **(E, G)** at D20 **(A, E)** or D30 **(C, G)** post-induction. Catalase/GAPDH protein expression was expressed as the relative protein expression (%) compared to CTL + vehicle group, in the brain **(B, D)** and the spinal cord **(F, H)** at D20 **(B, F)** or D30 **(D, H)** post-induction. For each protein level quantification, a representative Western blotting image of the target protein and loading control is proposed. Statistical analysis was performed using one-way ANOVA followed by Tukey’s multiple comparison test: *p < 0.05, **p < 0.01, ***p < 0.001 EAE + vehicle vs CTL + vehicle; #p < 0.05, ##p < 0.01, ###p < 0.001 EAE + GSE vs EAE + vehicle and $p < 0.05, $$$p < 0.001 EAE + vehicle vs CTL + GSE.

Catalase activity decreased in EAE mouse brains by 53% at D20 Tukey’s test, p < 0.001, and 56% at D30 Tukey’s test, p < 0.01, and was significantly corrected by GSE (**Figures 5A, C**). Similar results were found in the spinal cord where CAT activity decreased in EAE mice (59% at D20 Tukey’s test, p < 0.001, and 58% at D30 Tukey’s test, p < 0.01), which was significantly corrected upon GSE treatment (**Figures 5E, G**). Catalase protein expression decreased in EAE mouse brains by 56% at D20 (Tukey’s test, p < 0.001) and 41% at D30 (Tukey’s test, p < 0.05), which was significantly corrected upon GSE treatment (**Figures 5B, D**). Catalase protein expression significantly decreased in EAE mice spinal cord at D20 (50%, Tukey’s test, p < 0.001) with a trend at D30 (26%). A significant corrective effect upon GSE treatment was detected only at D20 (**Figures 5F, H**). Taken together, these data suggest a decrease in both CAT activity and expression from the peak of symptom to the chronic phase in the brain and spinal cord of EAE mice with a corrective effect of GSE that was more obvious in the brain than spinal cord compartment.

#### 3.6.3 Glutathione peroxidase activity and protein expression

**Figure 6:**
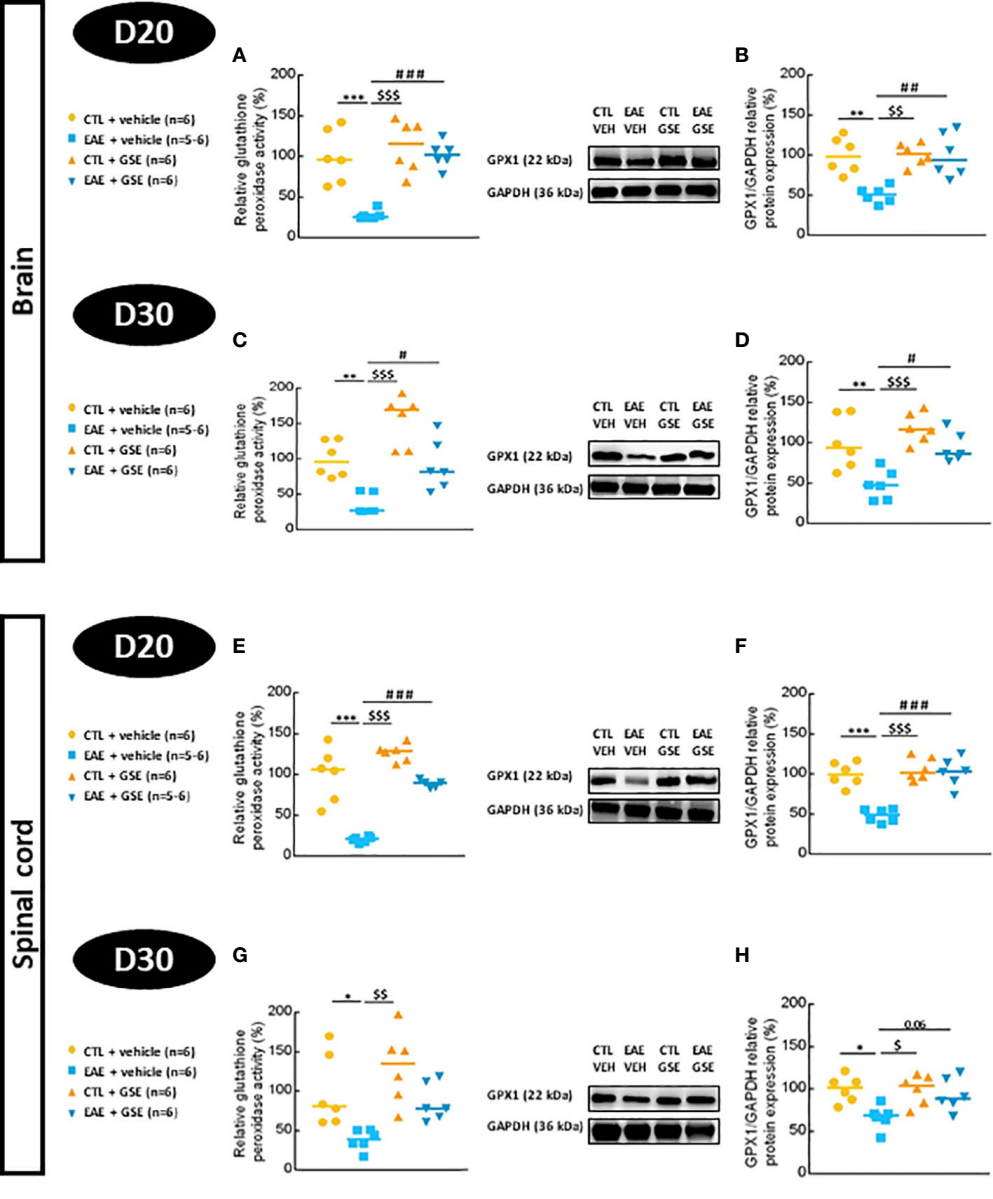
Evaluation of glutathione peroxidase activity and protein expression in the brain and spinal cord of experimental autoimmune encephalomyelitis mice (EAE) and their controls (CTL) after curative treatment with grape seed extract (GSE) or vehicle. Glutathione peroxidase activity was expressed as the relative activity (%) compared to CTL + vehicle group, in the brain **(A, C)** and the spinal cord (E, G) at D20 **(A, E)** or D30 **(C, G)** post-induction. GPX1/GAPDH protein expression was expressed as the relative protein expression (%) compared to CTL + vehicle group, in the brain **(B, D)** and the spinal cord **(F, H)** at D20 **(B, F)** or D30 **(D, H)** post-induction. For each protein level quantification, a representative Western blotting image of the target protein and loading control is proposed. Statistical analysis was performed using one-way ANOVA followed by Tukey’s multiple comparison test: *p < 0.05, **p < 0.01, ***p < 0.001 EAE + vehicle vs CTL + vehicle; #p < 0.05, ##p < 0.01, ###p < 0.001 EAE + GSE vs EAE + vehicle and $p < 0.05, $$p < 0.01, $$$<0.001 EAE + vehicle vs CTL + GSE.

Glutathione peroxidase activity decreased in EAE mouse brains by 72% at D20 (Tukey’s test, p < 0.001) and 70% at D30 (Tukey’s test, p < 0.01), which was significantly corrected upon GSE treatment (**Figures 6A, C**). Similar results were found in the spinal cord where GPx activity decreased in EAE mice (80% at D20 Tukey’s test, p < 0.001 and 62% at D30 Tukey’s test, p < 0.05), and GSE significantly corrected this alteration only at D20 (**Figures 6E, G**). In the brain, GPX1 expression decreased in EAE mice (49% at D20 and 52% at D30, Tukey’s test, p < 0.01) and was significantly corrected upon GSE treatment (**Figures 6B, D**). In the spinal cord, a decrease in GPX1 expression was observed in EAE mice (52% at D20 Tukey’s test, p < 0.001 and 33% at D30 Tukey’s test, p < 0.05) with GSE significant restoring effect only at D20 even if a trend was observed at D30 (**Figures 6F, H**). Taken together, these data suggest a decrease in GPx activity related to a decrease in F.GPX1 expression from the peak of symptom to the chronic phase in both the brain and spinal cord of EAE mice with a corrective effect of GSE more significant in the brain than the spinal cord.

### 3.7 Grape seed extract treatment restores myelin and sirtuin protein expression and decreases astroglial and microglial protein expression in both brain and spinal cord

#### 3.7.1 Myelin protein expression

**Figure 7:**
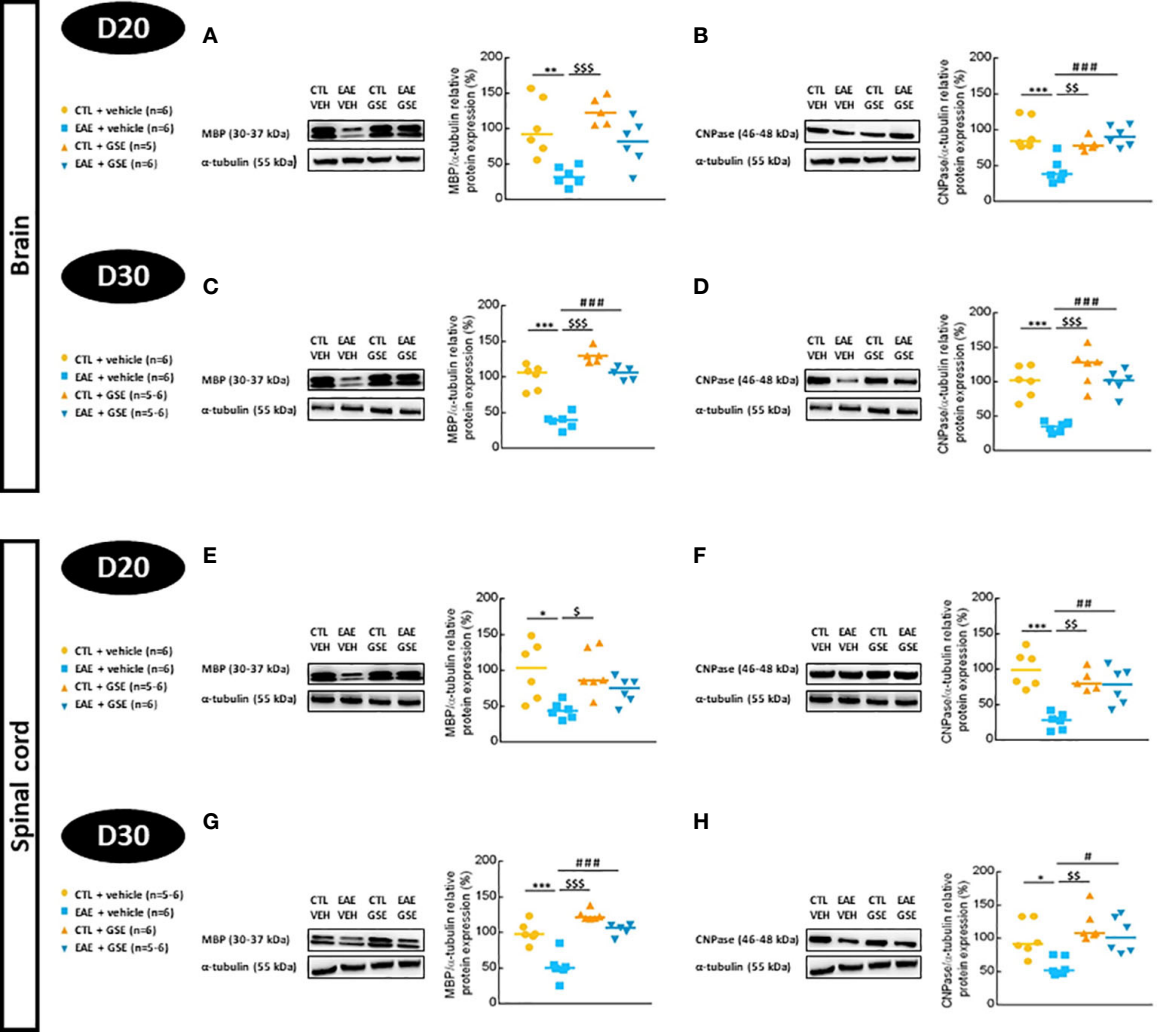
Evaluation of myelin basic protein (MBP) and 2′,3′-cyclic-nucleotide-3′-phosphatidesterase (CNPase) expressions in the brain and spinal cord of experimental autoimmune encephalomyelitis mice (EAE) and their controls (CTL) after curative treatment with grape seed extract (GSE) or vehicle. MBP/α-tubulin protein expression was expressed as the relative protein expression (%) compared to CTL + vehicle group, in the brain **(A, C)** and the spinal cord **(E, G)** at D20 **(A, E)** or D30 **(C, G)** post-induction. CNPase/α-tubulin protein expression was expressed as the relative protein expression (%) compared to CTL + vehicle group, in the brain **(B, D)** and the spinal cord **(F, H)** at D20 **(B, F)** or D30 **(D, H)** post-induction. For each protein level quantification, a representative Western blotting image of the target protein and loading control is proposed. Statistical analysis was performed using one-way ANOVA followed by Tukey’s multiple comparison test: *p < 0.05, **p < 0.01, ***p < 0.001 EAE + vehicle vs CTL + vehicle; #p < 0.05, ##p < 0.01, ###p < 0.001 EAE + GSE vs EAE + vehicle and $p < 0.05, $$p < 0.01, $$$p < 0.001 EAE + vehicle vs CTL + GSE.

Expression of MBP decreased in EAE mouse brains (67% at D20 Tukey’s test, p < 0.01 and 62% at D30 Tukey’s test, p < 0.001) (**Figures 7A, C**) and spinal cord (52% at D20 Tukey’s test, p < 0.05 and 48% at D30 Tukey’s test, p < 0.001) (**Figures 7E, G**), and GSE significantly corrected these alterations only at D30. Similarly, CNPase expression decreased in EAE mouse brains (57% at D20 and 66% at D30, Tukey’s test, p < 0.001) (**Figures 7B, D**) and spinal cord (73% at D20 Tukey’s test, p < 0.001 and 42% at D30 Tukey’s test, p < 0.05) (**Figures 7F, H**) with GSE significantly correcting these alterations at D20 and D30. These data suggest a decreased expression of two major CNS myelin proteins: MBP and CNPase in the brain and spinal cord of EAE mice from the peak of symptoms to the chronic phase of the disease. Importantly, such alterations were corrected upon GSE treatment at both phases of the disease for CNPase but only at the later phase for MBP.

#### 3.7.2 Astroglial and microglial protein expression

**Figure 8:**
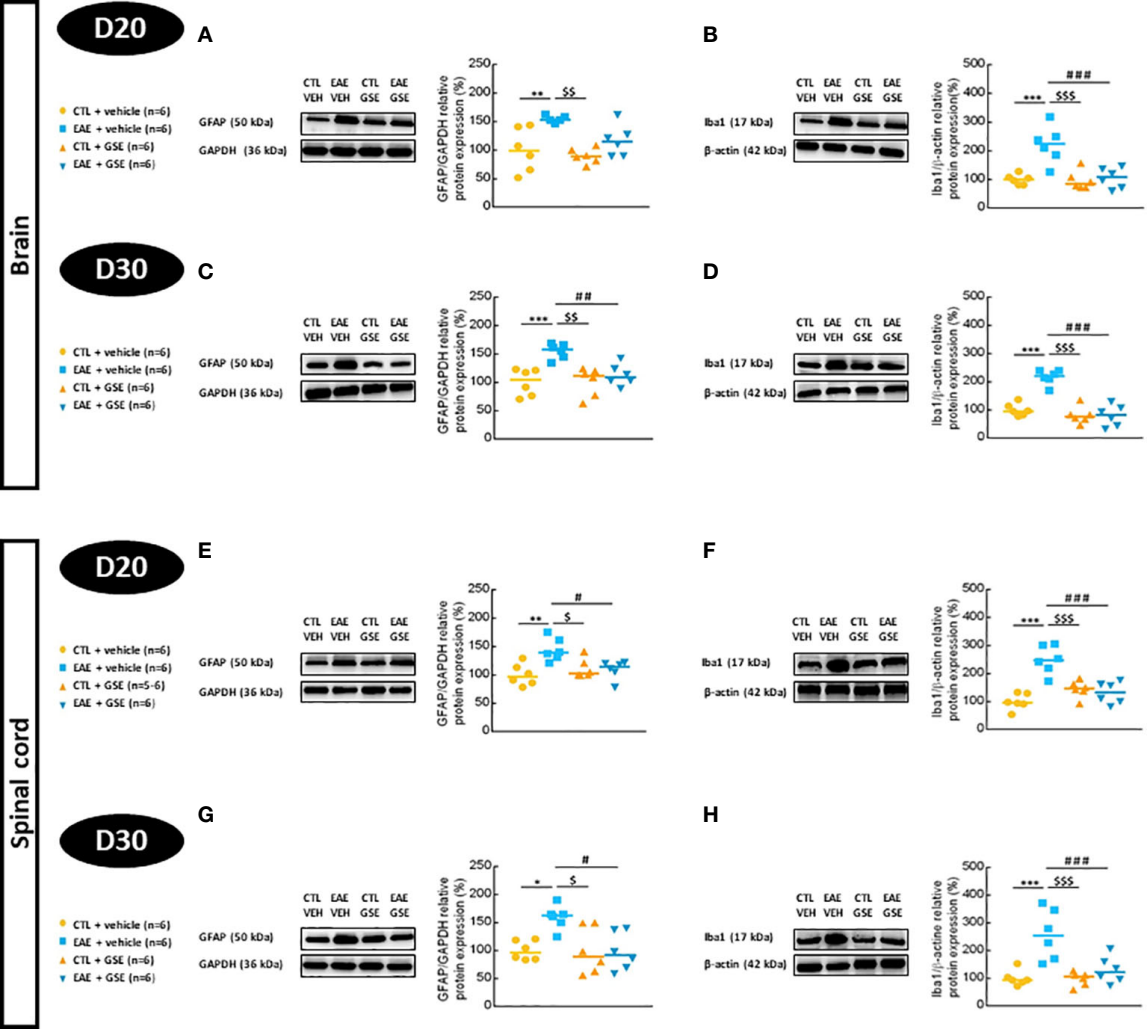
Evaluation of glial fibrillary acidic protein (GFAP) and ionized calcium-binding adaptor molecule 1 (Iba1) protein expressions in the brain and spinal cord of experimental autoimmune encephalomyelitis mice (EAE) and their controls (CTL) after curative treatment with grape seed extract (GSE) or vehicle. GFAP/GAPDH protein expression was expressed as the relative protein expression (%) compared to CTL + vehicle group, in the brain **(A, C)** and the spinal cord **(E, G)** at D20 **(A, E)** or D30 **(C, G)** post-induction. Iba1/β-actin protein expression was expressed as the relative protein expression (%) compared to CTL + vehicle group, in the brain **(B, D)** and the spinal cord **(F, H)** at D20 **(B, F)** or D30 **(D, H)** post-induction. For each protein level quantification, a representative Western blotting image of the target protein and loading control is proposed. Statistical analysis was performed using one-way ANOVA followed by Tukey’s multiple comparison test: *p < 0.05, **p < 0.01, ***p < 0.001 EAE + vehicle vs CTL + vehicle; #p < 0.05, ##p < 0.01, ###p < 0.001 EAE + GSE vs EAE + vehicle and $p < 0.05, $$p < 0.01, $$$p < 0.001 EAE + vehicle vs CTL + GSE.

In the brain, GFAP expression increased in EAE mice (47% at D20 Tukey’s test, p < 0.01 and 55% at D30 Tukey’s test, p < 0.001), and GSE significantly corrected this alteration only at D30 (**Figures 8A, C**). In the spinal cord, GFAP expression also increased in EAE mice (49% at D20 Tukey’s test, p < 0.01, and 59% at D30 Tukey’s test, p < 0.05), and GSE significantly corrected these alterations (**Figures 8E, G**). Expression of Iba1 significantly increased in EAE mouse brains (121% at D20 and 116% at D30, Tukey’s test, p < 0.001) (**Figures 8B, D**) and spinal cord (149% at D20 and 158% at D30, Tukey’s test, p < 0.001) (**Figures 8F, H**). Curative treatment with GSE significantly corrected these alterations. Taken together, these data suggest an increase in astroglial and microglial protein expression in the brain and spinal cord of EAE mice from the peak of symptoms to the chronic phase, which was corrected upon GSE treatment at earlier and later phases for microglial protein and at a later phase for astroglial protein.

#### 3.7.3 Sirtuin protein expression

**Figure 9:**
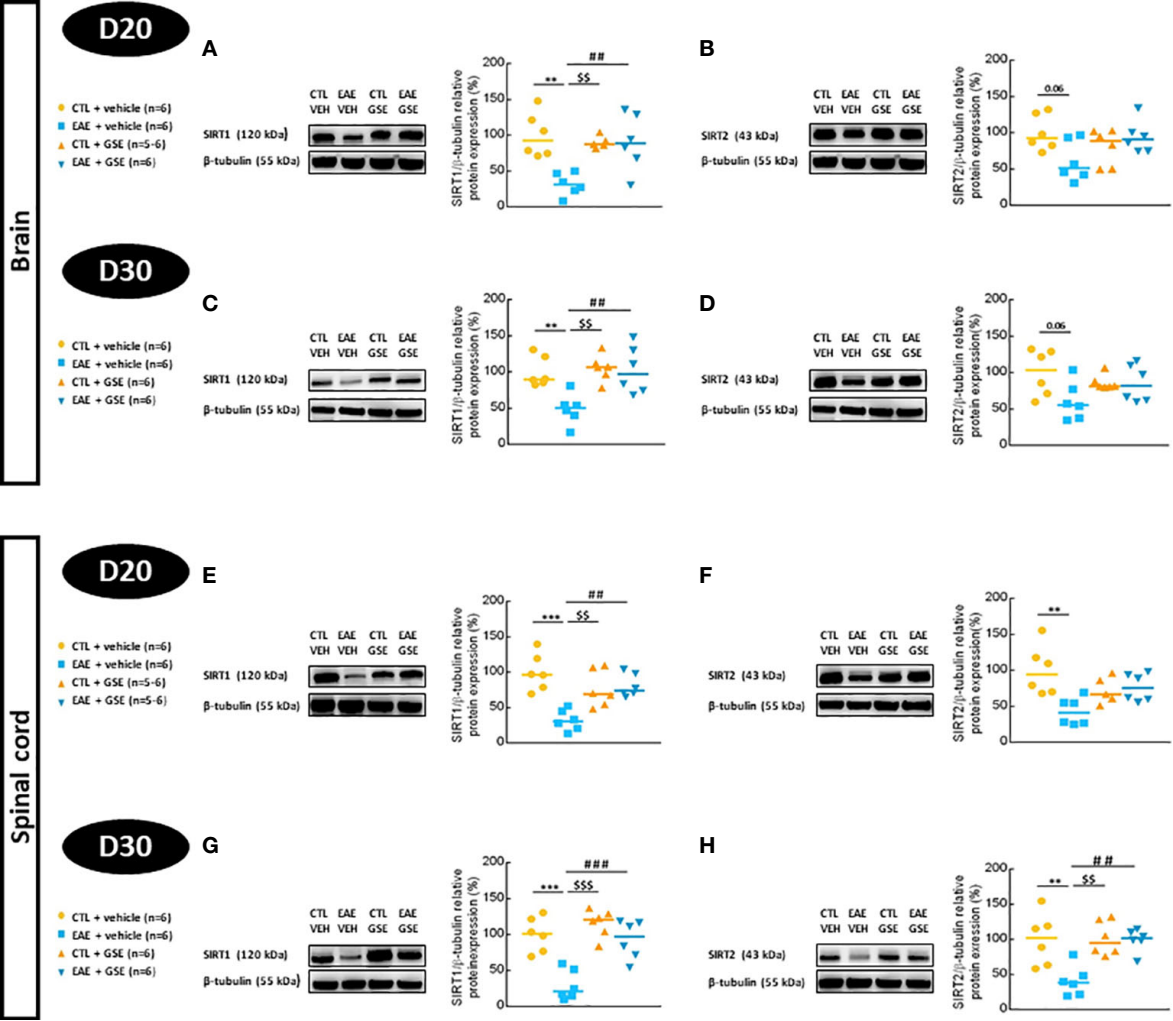
Evaluation of sirtuin 1 (SIRT1) and sirtuin 2 (SIRT2) protein expressions in the brain and spinal cord of experimental autoimmune encephalomyelitis mice (EAE) and their controls (CTL) after curative treatment with grape seed extract (GSE) or vehicle. SIRT1/β-tubulin protein expression was expressed as the relative protein expression (%) compared to CTL + vehicle group, in the brain **(A, C)** and the spinal cord **(E, G)** at D20 **(A, E)** or D30 **(C, G)** post-induction. SIRT2/β-tubulin protein expression was expressed as the relative protein expression (%) compared to CTL + vehicle group, in the brain **(B, D)** and the spinal cord **(F, H)** at D20 **(B, F)** or D30 **(D, H)** post-induction. For each protein level quantification, a representative Western blotting image of the target protein and loading control is proposed. Statistical analysis was performed using one-way ANOVA followed by Tukey’s multiple comparison test: **p < 0.01, ***p < 0.001 EAE + vehicle vs CTL + vehicle; ##p < 0.01, ###p < 0.001 EAE + GSE vs EAE + vehicle and $$p < 0.01, $$$p < 0.001 EAE + vehicle vs CTL + GSE.

The expression of SIRT1 significantly decreased in EAE mouse brains (68% at D20 and 69% at D30, Tukey’s test, p < 0.01) (**Figures 9A, C**) and spinal cord (68% at D20 and 70% at D30, Tukey’s test, p < 0.001) (**Figures 9E, G**), and GSE significantly corrected such alterations. Regarding SIRT2 expression, no statistical difference between groups was detected in the brain at both times of evaluation (Tukey’s test, p > 0.05) (**Figures 9B, D**). In the spinal cord, SIRT2 decreased in EAE mice (56% at D20 and 59% at D30, Tukey’s test, p < 0.01), and GSE significantly corrected this alteration only at D30 (**Figures 9F, H**). These data suggest a decreased expression of sirtuins from the peak of symptoms to the chronic phase of the disease in both the brain and spinal cord for SIRT1 and only in the spinal cord for SIRT2. Treatment with GSE restored the level of both sirtuins as soon as 10 days of treatment for SIRT1 and far away from 10 days for SIRT2.

## 4 Discussion

We investigated the corrective effect of chronic GSE treatment in an EAE mouse model. Our results showed that GSE counterbalanced the EAE clinical development and corrected mechanical and cold allodynia. Data from brain and spinal cord demonstrated that GSE reduced oxidative stress damage (lipid peroxidation and protein carbonylation) by restoring antioxidant capacities (increase in SOD, CAT, and GPx enzymes activities and protein expressions). This antioxidative effect led to the restoration of normal MBP and CNPase protein expression as well as correction of microglial Iba1 and astroglial GFAP protein overexpression and SIRT1 and SIRT2 protein downexpression.

As described by Olechowski et al. (2009), in our EAE model, symptoms began as paralysis in the tail at D9 (clinical grade 1) and progressed to more severe clinical deficits (grade 3 or 4) by D21. Chronic curative GSE treatment initiated at D10 reduced the development of the EAE clinical course as soon as D14 to a full correction observed since D25. We chose its EAE model, as it develops robust mechanical allodynia prior to and at the onset of neurological deficits and cold allodynia occurring from the peak of symptoms. In our study, we replicated chronologies of symptoms and showed that GSE treatment corrected the development of mechanical hypersensitivity and prevented the development of cold allodynia. In order to evaluate sensitive dysfunction, motor dysfunctions occurring following the immunization process should be reduced (16, 30). As such, in the present study, no alteration of spontaneous locomotor activity nor motor coordination was observed, nor did GSE alter these activities. Given that pain in MS showed greater interference with fatigue, depression, anxiety, and cognitive impairment (5, 31), a broad evaluation of EAE-induced behavioral disabilities is of utmost importance. Conversely to the literature, where a defect in novel object recognition (30) and deficit of hippocampal memory (32, 33) were demonstrated in similar EAE models, we failed to observe any defect in working memory using the Y-maze test. We also failed to record any alteration in exploring related anxiety behaviors using either EPM (34) or open-field test previously observed in a similar EAE model (35, 36). Such discrepancy with data from literature could be explained by the difference in the immunization protocol, behavioral tests used, or timing of evaluation. Future studies using different timing for behavioral evaluations or other variants of the EAE or non-EAE MS mouse model are then warranted to evaluate the GSE effect on cognitive dysfunctions and anxiety-related behaviors.

Multiple sclerosis is a multifactorial disease in which oxidative stress is a worsening factor in disease progression. We showed in both the brain and spinal cord an increase in lipid peroxidation disappearing during the chronic phase of the disease, while protein carbonylation culminated at both phases of the disease; these defects were corrected by GSE as soon as 10 days of treatment in both tissues. These oxidative processes were associated with a decrease in enzymatic activities and protein expressions of the first line of antioxidant intracellular defense, namely, cytosolic SOD1 and mitochondrial SOD2, which remove O_2_^•−^ by producing H_2_O_2_ as well as CAT and GPXs, which eliminate H_2_O_2_ (36). Previous studies showed altered antioxidant enzyme activities in the brain of another CFA EAE mouse model, during the chronic phase of the disease (37, 38). However, to our knowledge, our study is the first to evaluate the enzymatic activity of the three main antioxidant enzymes in EAE mice: i) during the early and late stages of the disease development and ii) in both the brain and spinal cord. Treatment with GSE clearly improves SOD, CAT, and GPx activities, as well as protein expression in both the brain and spinal cord as soon as 10 days of treatment; it is tempting to speculate on the beneficial effect of GSE in MS patients. In fact, it has already been established that antioxidant enzyme levels dropped drastically during the course of the disease (39). Very strikingly, oxidative defects corrected by GSE in EAE mice are similar to the presence of lipid peroxides or their breakdown products such as MDA, oxidized phospholipids, and DNA, which have been largely described in MS lesions (39, 40). Finally, this study did not evaluate the production of reactive species nor the aberrant redox signaling leading to oxidized macromolecules due to the choice of our therapeutic protocol. Treatment with GSE was initiated at D10, when the EAE development was already well ongoing (first symptoms are already observed). We then considered the characterization of the first step of oxidative stress development in EAE mice beyond the scope of this article. However, the evaluation in the near future of the effect of GSE (using earlier treatment initiation) on the production of reactive species and aberrant redox signaling would be of huge interest.

As a hallmark of MS pathology, the demyelination process was assessed by Western blotting quantification of MBP and CNPase protein expression. Our data fully support CNS demyelination during the acute and chronic stages of EAE, in support of literature (41, 42). While GSE treatment corrected CNPase expression as soon as 10 days of treatment in both the brain and spinal cord, significant correction of MBP alterations were observed only later. The faster corrective effect of GSE on CNPase could be linked to the abundance of such membrane-associated enzymes whose expression is sharply decreased in compromised oligodendrocytes but can be rapidly restored upon protective treatment (43). In contrast, MBP is a structural protein necessary for the maintenance of myelin sheath and myelination process, and its expression decreased strongly during the demyelination process, and as a result, efficient treatment should work on halting demyelination while favoring remyelination, which are time-consuming processes (44). In MS patients and EAE models, destruction of CNS myelin is associated with activated microglia and astrocytes, which are involved in disease pathogenesis. As expected, we showed an increase in microglial (Iba1) and astroglial (GFAP) protein expression in the brain and spinal cord of EAE mice. According to the literature, Iba1 overexpression was of higher amplitude than GFAP overexpression (45), confirming that microglia plays a major role in the physiopathology of EAE (46, 47). Very interestingly, GSE chronic treatment was able to correct overexpression of Iba1 in the early and late phases of the disease and GFAP in the late phase. Finally, we showed an early and long-lasting decrease in SIRT1 expression in the brain and spinal cord of EAE mice, while a decrease in SIRT2 expression was limited to the spinal cord. Our data are in accordance with the altered expression of SIRT2 depicted in an EAE model and *post-mortem* brain lesions (48). Involvement of SIRT1 in EAE mice is more controversial, as many studies described either deleterious effects when expressed in peripheral immune cells (49) or beneficial effects when expressed in central tissues (50, 51). Although data are rather scarce in MS patients, they suggest a deleterious effect of low SIRT1 expression (52). Interestingly, GSE treatment corrected both SIRT1 and SIRT2 downexpression, which could be explained by the presence of quercetin (19), a robust inhibitor of the NAD^+^ase CD38, increasing cellular NAD^+^ availability and reducing protein acetylation (53). Our data are far too limited to the precise involvement of sirtuins in EAE pathology but are part of an increasing amount of data suggesting that sirtuins are compromised in autoimmune and neurological diseases and their relevance as therapeutic targets (54, 55).

The wide range of GSE effects on EAE physiopathology described in the present study is likely due to the presence of a large number of bioactive compounds and their synergism. We already described how quercetin could be involved in the upregulation of sirtuins. According to previous data, quercetin could also decrease myelin phagocytosis and then limit the demyelination process during MS (56). Finally, and probably being its main effect regarding results of this study, quercetin is a strong nuclear factor erythroid-2-related factor 2 (Nrf2) activator and as such is undoubtedly involved in the induction of antioxidant enzymes (36). The abundance of gallic acid, catechin, and epicatechin is also relevant to GSE composition and could support the involvement of these bioactive molecules in the beneficial effect observed in this study (19). Limitations of antioxidant therapy have been recently discussed by Forman and Zhang (2021), and among these major concerns one can evoke: “The claim that an antioxidant is a •OH scavenger is meaningless as almost all molecules react with •OH at about the same rate, thus, the only defense against •OH is to prevent its formation, and the most effective way to achieve that is H_2_O_2_ elimination.” Hence, compounds with SOD and CAT enhancing activity or ability to induce the activity of biosynthetic pathway enzymes are of great interest. Although oxidative stress is a secondary contributor to MS development, drugs with antioxidant effects have already shown their efficacy and are already used in MS patients, as exemplified by dimethyl fumarate (57). Hence, due to its pleiotropic mechanisms of action, its strong effect in EAE mice described in this study, and its very good safety observed in the first clinical studies (9, 10), GSE harbored a high therapeutic potential for MS treatment.

Finally, regarding the way of administration, we made the choice to use intraperitoneal injection (rather than the oral route) in order to improve polyphenol bioavailability, limiting their biotransformation by digestive enzymes and hence showed a very strong effect on GSE in our EAE model. In anticipation of a possible clinical indication of GSE, it will be important to develop new formulations able to achieve long-term treatments with high doses of polyphenols with an important bioavailability for CNS tissues.

## 5 Conclusion

Our study focused on the curative effect of GSE when early neurological symptoms (EAE score and mechanical allodynia) are detected and when the immune and inflammatory processes have already spread from the periphery to the CNS. Hence, the therapeutic protocol herein did not allow studying the putative effect of GSE on early lymphoid immune response or the early stage of CNS pathology, and as a result, no immunological evaluation was undertaken. Investigating the mechanism of action of GSE, our study revealed that GSE alleviated oxidative stress partly *via* its antioxidant properties as well as through several other cellular signaling pathways including modulation of myelin, microglia, astrocytes, and sirtuin protein expression, emphasizing GSE as a safe and highly promising pharmacological agent for MS treatment. This preliminary work paved the way for future studies investigating 1) the early cellular and molecular pathways responsible for GSE-induced EAE suppression, 2) the curative effect of GSE on other symptoms than EAE score and sensitive behaviors using various EAE and non-EAE models of MS, and 3) the preventive effect of GSE. However, the huge effect induced by GSE curative treatment on EAE score and sensitive alterations (symptoms yet highly resistant to current treatment) strongly supports GSE as an effective therapeutic approach for treating MS.

## Data availability statement

The raw data supporting the conclusions of this article will be made available by the authors, without undue reservation.

## Ethics statement

The animal study was reviewed and approved by the local ethic committee C2EA-02 (Authorization delivered by the Minister of Higher Education, Research and Innovation APAFIS-4306).

## Author contributions

MMa performed the experiments, derived the models, analyzed the data and wrote the first draft of the manuscript; ME provided technical support and discussed the results; AD provided technical support and contributed to the final manuscript; YA provided technical support and contributed to the final manuscript; EA discussed the results and contributed to the final manuscript; L-DT discussed the results and contributed to the final manuscript; MMo supervised the project, discussed the results and contributed to the final manuscript; MB designed and directed the project, contributed to data or analysis tools and participated to all the step of the manuscript preparation. All authors contributed to the article and approved the submitted version.

## Funding

This work was financially supported by the ‘PHC Utique’ program of the French Ministry of Foreign Affairs and Ministry of Higher Education, Research and Innovation and the CMCU project number 20G0803. MM’s PhD training was supported by the ‘Bourse d’alternance’ from the Tunisian Ministry of Higher Education and Scientific Research and the Tunis El Manar University as well as the ‘WOW (Wide Open to the World) CAP2-25 program’ of the UCA University. This work was also partly supported by the Ministère Français de l’Enseignement Supérieur et de la Recherche (AD PhD fellowship), ARSEP Foundation (AAP 2014), and French government IDEX-ISITE initiative (grant number 16-IDEX-0001-CAP 20-25) of the University of Clermont Auvergne.

## Conflict of interest

The authors declare that the research was conducted in the absence of any commercial or financial relationships that could be construed as a potential conflict of interest.

## Publisher’s note

All claims expressed in this article are solely those of the authors and do not necessarily represent those of their affiliated organizations, or those of the publisher, the editors and the reviewers. Any product that may be evaluated in this article, or claim that may be made by its manufacturer, is not guaranteed or endorsed by the publisher.
