# Supplementary figures and images for: Antioxidant effect of grape seed extract corrects experimental autoimmune encephalomyelitis behavioral dysfunctions, demyelination, and glial activation
Source: Front Immunol. 2022 Aug 17;13:960355. doi: 10.3389/fimmu.2022.960355 (PMC9428676; doi:10.3389/fimmu.2022.960355)

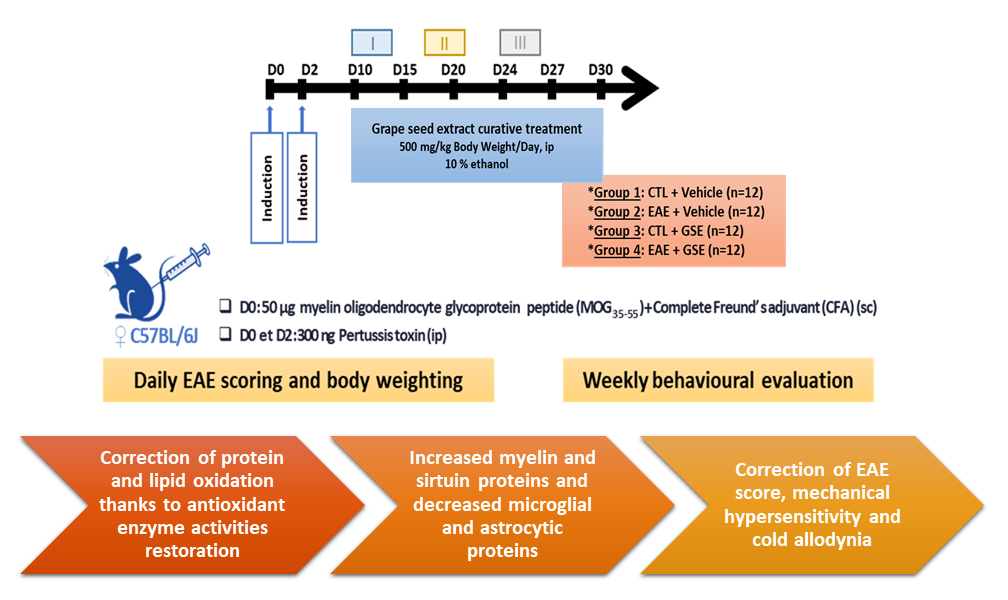

Supplement: Supplementary file 1 [file Image_1.tif]
